# Extracellular Vesicle Release from Immune Cells in Cutaneous Leishmaniasis: Modulation by *Leishmania (V.) braziliensis* and Reversal by Antimonial Therapy

**DOI:** 10.3390/pathogens14080771

**Published:** 2025-08-04

**Authors:** Vanessa Fernandes de Abreu Costa, Thaize Quiroga Chometon, Katherine Kelda Gomes de Castro, Melissa Silva Gonçalves Ponte, Maria Inês Fernandes Pimentel, Marcelo Rosandiski Lyra, Rienk Nieuwland, Alvaro Luiz Bertho

**Affiliations:** 1 Laboratory of Immunoparasitology, Oswaldo Cruz Institute, FIOCRUZ, Rio de Janeiro 21040-360, Brazil; vanessafcsta@gmail.com (V.F.d.A.C.); melissaponte@live.com (M.S.G.P.); 2 Flow Cytometry Core Facility, Oswaldo Cruz Institute, FIOCRUZ, Rio de Janeiro 21040-360, Brazil; thaizeqcp@gmail.com (T.Q.C.); katherine.castro@ioc.fiocruz.br (K.K.G.d.C.); 3 Laboratory of Clinical Research and Surveillance in Leishmaniasis, Evandro Chagas National Institute of Infectious Diseases, FIOCRUZ, Rio de Janeiro 21040-360, Brazil; maria.pimentel@ini.fiocruz.br (M.I.F.P.); marcelo.lyra@ini.fiocruz.br (M.R.L.); 4 Laboratory of Experimental Clinical Chemistry, Laboratory Specialized Diagnostics & Research, Department of Laboratory Medicine, Amsterdam UMC, University of Amsterdam, 1105 AZ Amsterdam, The Netherlands; r.nieuwland@amsterdamumc.nl; 5 Amsterdam Vesicle Center, Amsterdam UMC location University of Amsterdam, 1105 AZ Amsterdam, The Netherlands

**Keywords:** cutaneous leishmaniasis, *Leishmania (Viannia) braziliensis*, extracellular vesicles, nano-flow cytometry, immune cell-derived EV, immune biomarkers, lymphocyte proliferation assay, immunomodulation, host–parasite interaction, antimonial therapy

## Abstract

Human cutaneous leishmaniasis (CL) caused by *Leishmania (Viannia) braziliensis* is a complex parasitic disease marked by dynamic host–parasite interactions and immunomodulation. Extracellular vesicles (EV) derived from immune cells have emerged as key mediators of intercellular communication and potential biomarkers in infectious diseases. In this study, we combined a modified lymphocyte proliferation assay with nano-flow cytometry to quantify and phenotype EV released by CD4^+^, CD8^+^, and CD14^+^ cells in PBMC cultures from CL patients at different clinical stages: before treatment (PBT), during treatment (PDT), and post-treatment (PET) with antimonial. Healthy individuals (HI) were included as physiological controls. Upon stimulation with *L. (V.) braziliensis* antigens, we observed a distinct modulation of EV subsets. In the PBT group, CD4^+^ and CD14^+^ EV were significantly reduced, while CD8^+^ EV remained elevated. During PDT and PET, EV concentrations were restored across all subsets. These findings suggest that *L. (V.) brazilie*nsis selectively modulates the release of immune cell–derived EV, possibly as an immune evasion mechanism. The restoration of EV release following antimonial therapy highlights their potential as sensitive biomarkers for disease activity and treatment monitoring. This study offers novel insights into the immunoregulatory roles of EV in CL and underscores their relevance in host–parasite interactions.

## 1. Introduction

Cutaneous leishmaniasis (CL) is a vector-borne parasitic disease caused by protozoa of the genus *Leishmania*, transmitted to humans through the bite of infected phlebotomine sandflies. Globally, CL ranks among the top ten neglected tropical diseases and remains an important public health concern due to its wide distribution and persistent endemicity [1]. The disease is endemic in over 90 countries, with the high incidence in regions of the Middle East, South America, and Central Asia. In Brazil, CL is broadly distributed, and in the state of Rio de Janeiro, all cases are attributed to *Leishmania (Viannia) braziliensis*, with *Lutzomyia* species serving as the primary vector.

Clinically, CL typically presents as localized papules that progress to ulcerative skin lesions with raised, indurated borders and granulomatous base [2,3,4]. Although some lesions may resolve spontaneously, they often leave disfiguring scars with considerable psychosocial consequences. Standard treatment relies on pentavalent antimonial, such as meglumine antimoniate (MA) [2,3,4,5], although therapeutic alternatives remain limited.

Host immune response plays a pivotal role disease outcome. A robust CD4^+^ Th1 response, characterized by interleukin-2 (IL-2), interferon-γ (IFN-γ), and interleukin-12 (IL-12) production, promotes macrophage activation and parasite clearance, whereas Th2-skewed response, marked by interleukin-4 (IL-4), interleukin-10 (IL-10), and transforming growth factor-β (TGF-β), favors parasite persistence and lesion chronicity [6,7,8]. CD8^+^ T cells also contribute to parasite control via IFN-γ-release immune response but have been implicated in exacerbating tissue damage and lesion severity through cytotoxic mechanisms [9,10,11,12,13,14,15,16,17].

Intercellular communication plays a critical role in coordinating the immune response to *Leishmania*. Extracellular vesicles (EV)—membrane-bound nanoparticles released by nearly all cell types—have emerged as important mediators of immune signaling, antigen presentation, and cytokine transport [18,19,20,21,22,23]. These vesicles carry molecular cargoes, including proteins, lipids, and nucleic acids, which reflect the identity and functional state of their cell of origin.

To date, few translational studies have explored the involvement of immune cell–derived EV in the pathogenesis of cutaneous leishmaniasis (CL) caused by *L. (V.) braziliensis*. Most EV-related research in parasitic infections has focused primarily on parasite-derived vesicles and their impact on host cells [24,25,26,27]. Therefore, this study aimed to evaluate the dynamics of immune cell–derived EV in different clinical stages of CL and to assess the potential modulation of their release by *L. (V.) braziliensis* infection and antimonial therapy.

Our results reveal a distinct biphasic pattern in EV dynamics over the course of infection, characterized by a reduction in EV release from CD4^+^ T cells and CD14^+^ monocytes during the active phase, followed by a significant resurgence during and after antimonial therapy. These findings not only advance our understanding of EV-mediated immunomodulation in CL, but also establish a foundation for future longitudinal and tissue-based investigations to elucidate the mechanistic roles of EV in CL pathophysiology.

## 2. Materials and Methods

### 2.1. Study Cohorts

This study was a longitudinal study with a total of 37 patients diagnosed for cutaneous leishmaniasis (CL) by at least one of the following methods: parasite isolation in NNN medium supplemented with Schneider’s Drosophila medium [2], direct detection of parasites by light microscopy observation after Giemsa staining [2], histopathologic analysis of the inflammatory infiltrate [28], or PCR targeting kinetoplast DNA [29]. The species of isolated parasites were characterized by isoenzyme electrophoresis profiles [30]. All of the 37 patients, residents in endemic areas of Rio de Janeiro, Brazil, who voluntarily agreed to participate in this project, signed, before blood collection, the Free and Informed Consent Form, written in accordance with the norms of Resolution 466/2012 of the National Health Council, Ministry of Health, Brazil. Moreover, they were treated and followed up at the Leishmaniasis Surveillance Laboratory, Evandro Chagas National Institute of Infectious Diseases (INI), Oswaldo Cruz Foundation (FIOCRUZ), Rio de Janeiro, Brazil.

Exclusion criteria included the following: age below 18 or above 60 years; pregnancy; comorbidities such as HIV/AIDS, COVID-19, Chagas disease, malaria, diabetes, autoimmune disorders, or viral hepatitis; prior treatment for leishmaniasis; and other coexisting dermatological conditions. None of the patients developed mucocutaneous or disseminated forms of the disease. All patients received intramuscular meglumine antimoniate (MA) (Glucantime^®^, Aventis, Paris, France) at a dose of 5 mg Sb^5+^/kg/day for 30 consecutive days, limited to a maximum daily dose of 1215 mg Sb^5+^ which is equivalent to 3 MA vials or 15 mL [2,5].

Patients were stratified into three clinical groups based on treatment status: (i) PBT (patients before treatment; *n* = 17), corresponding to individuals in the acute phase of disease; (ii) PDT (patients during treatment; *n* = 10), assessed between days 10 and 13 of antimonial therapy; and (iii) PET (patients at the end of treatment; *n* = 10), examined between days 70 and 95 after the beginning of treatment, and classified as clinically cured. Clinical cure was defined as the complete resolution of cutaneous lesions and clinical symptoms, with no signs of active infection. An additional group of six healthy individuals (HI) without any history of leishmaniasis and residing in non-endemic areas of Rio de Janeiro was included as a control.

The main clinical features of the studied subjects are described in Table 1. Among patients, 54.05% were male, and the mean±SD age was 36.2 ± 15.1 years. The number of lesions per patient ranged from 1 to 4, and lesion diameters ranged from 14 to 130 mm. The six healthy individuals had a mean age of 30 ± 10 years and an equal sex distribution (50% male, 50% female).

### 2.2. Reagents

For the lymphocyte proliferation assay (LPA), RPMI 1640 cell culture medium, fetal bovine serum (FBS), Ca^2+^/Mg^2+^-free Dulbecco’s phosphate-buffered saline (DPBS), Ca^2+^/Mg^2+^-free Hanks’ balanced salt solution (HBSS) (all from Thermo Fisher Scientifics, Waltham, MA, USA), HEPES, L-glutamine, penicillin, streptomycin, and 2-mercaptoethanol (all from Sigma Aldrich Brasil Ltda, Cotia, Brazil, were used. The reagents used to define and phenotype EV included annexin V-FITC (BD Biosciences, São Paulo, Brazil); calcein violet AM (BioLegend, San Diego, CA, USA); monoclonal antibodies targeting CD3-PECy7, CD4-APC, CD8-PECy5.5, and CD14-PE (Beckman Coulter, Brea, CA, USA); and IgG1 mouse isotype controls (Beckman Coulter, São Paulo, Brazil)). Additional materials included annexin V binding buffer (BD Biosciences, São Paulo, Brazil) and polystyrene calibration Megamix-Plus FSC and SSC beads (BioCytex, Marseille, France) for EV size correlation. Additionally, 0.22 µm-filtered Milli-Q water was utilized as flow cytometer sheath fluid, while DNAse, RNAse-free UltraPure™ distilled water (Life Technologies/Invitrogen, Grand Island, NY, USA) served for quality control and cleaning flow cytometry fluidics between sample acquisitions.

### 2.3. Removal of Endogenous Extracellular Vesicles from RPMI 1640 Medium and Fetal Bovine Serum (FBS)

To minimize potential interference from naturally occurring extracellular vesicles (EV) present in the RPMI 1640 and fetal bovine serum (FBS) used in the lymphocyte proliferation assay (LPA), EV-depleted medium was prepared as follows. RPMI 1640 culture medium (Thermo Fisher Scientifics, Waltham, MA, USA), supplemented with 10 mM HEPES, 1.5 mM L-glutamine, 200 IU/mL penicillin, 200 μg/mL streptomycin, 0.04 mM 2-mercaptoethanol, and 20% FBS (all from Sigma Aldrich Brasil Ltda, Cotia, Brazil), was transferred into Quick-Seal round-top polypropylene ultracentrifuge tubes (Beckman Coulter, Indianapolis, IN, USA) and sealed according to the manufacturer’s instructions. The tubes were subjected to ultracentrifugation at 100,000× *g* for 18 h at 4 °C using a 70.1 Ti rotor in an Optima L-100 XP ultracentrifuge (Beckman Coulter, São Paulo, Brazil) [31]. Following ultracentrifugation, the supernatant was carefully collected using a syringe and needle, with the tube held at an angle to avoid disturbing the EV-containing pellet; approximately 0.5 cm of the supernatant above the pellet was left undisturbed. The collected medium was subsequently filtered through a 0.22 μm membrane filter (Millex-GV; Millipore, Cork, Ireland) and stored at 4 °C for a maximum of six months. The resulting EV-depleted FBS-supplemented RPMI 1640 medium (referred as UC-RPMI/FBS) was used in place of untreated FBS-containing RPMI 1640 medium in both the LPA and PBMC cryopreservation protocols.

### 2.4. Blood Collection

Blood collections were carried out at the LVL, INI, FIOCRUZ, RJ, Brazil, and carried out following the protocol established by Costa et al. (2024) [32]: 20 mL of peripheral blood from individuals in each group studied were collected by venipuncture, with Vacutainer™ (Becton Dickinson, Plymouth, UK) 21 gauge needles, without the use of a tourniquet and without agitation, to avoid the formation of EV during the process. The first tube of blood (approx. 2 mL) was discarded and 2 tubes of 10 mL containing sodium heparin (BD Biosciences, São Paulo, Brazil) were subsequently connected to the Vacutainer™ (Becton Dickinson, Plymouth, UK). The maximum time between the collection of biological material and its processing was 24 h; during this period, the tubes remained at room temperature (20 ± 2 °C), in an orbital shaker under homogenization (Arsec, São Paulo, Brazil).

### 2.5. Peripheral Blood Mononuclear Cell Cryopreservation Protocol

Peripheral blood mononuclear cells (PBMCs) were separated from the blood samples in Ficoll-Hypaque gradients (Histopaque-1077, Sigma, Cotia, Brazil), resuspended in RPMI 1640 supplemented with 10 mM HEPES, 1.5 mM/L L-glutamine, 200 IU/mL penicillin, 200 μg/mL streptomycin, and 0.04 mM 2-mercaptoethanol (all Sigma, Cotia, Brazil), adjusted to 1 × 10^7^ cells/mL, and preserved in a freezing solution (90% heat-inactivated UC-RPMI/FBS and 10% dimethyl sulfoxide) in cryotubes (Nunc™ Biobanking and Cell Culture Cryogenic Tubes, Thermo Fisher Scientific, Waltham, MA, USA). The cryotubes were initially frozen at −80 °C for 24 h before being transferred to liquid nitrogen for long-term storage.

### 2.6. Preparation of L. (V.) braziliensis Promastigotes Extract

The *L. (V.) braziliensis* (MCAN/BR/98/R619) promastigotes extract *(Lb*Ag) was generated according to Cunha et al. (2020) [13], with some modifications. Briefly, *L. (V.) braziliensis* promastigotes were cultured in Schneider medium supplemented with antibiotics (200 IU penicillin and 200 μg streptomycin/mL) and 10% inactivated UC-RPMI/FBS (all from Sigma, St. Louis, MO, USA), and 2% human urine (male donor), at 27 °C for 5 days. Stationary phase promastigotes were washed three times in DPBS, and disrupted by 10 freeze and thaw cycles, followed by ultrasonication (Ultra-tip Labsonic System; Laboratory-Line, Melrose Park, IL, USA), at 40 watts for 15 min in an ice bath. The antigen extract was 10 µL aliquoted and stored at −20 °C until use.

### 2.7. Lymphocyte Proliferation Assay and Harvesting of Extracellular Vesicles from PBMC Culture Supernatants

To enable the quantification and phenotyping of extracellular vesicles (EV) in peripheral blood mononuclear cells (PBMCs) culture supernatants, we modified the standard lymphocyte proliferation assay (LPA) protocol previously described [13]. Briefly, frozen PBMCs (prepared as described in Section 2.5) were rapidly thawed in a 37 °C water bath (VWR, Radnor, PA, USA), diluted in 1 mL of RPMI 1640 medium, and washed twice with Dulbecco’s phosphate-buffered saline (DPBS; 300× *g*, 4 °C, 10 min). The resulting cell pellet was resuspended in 1 mL of UC-RPMI/FBS (supplemented RPMI 1640 medium described in Section 2.3), cell viability was measured and accepted with viable cell rates higher than 80%, and cell concentration was adjusted to 5 × 10^5^ cells/100 µL of UC-RPMI/FBS. Aliquots of 100 µL (5 × 10^5^ cells) were plated in duplicate into 24-well flat-bottom polystyrene plates (Falcon®, BD Biosciences, San Jose, CA, USA). For stimulation, either 10 µg of *L. (V.) braziliensis* antigens (*Lb*Ag; final concentration 50 µg/mL) or 20 µg/mL of phytohemaglutinin-A (PHA; Sigma-Aldrich, St. Louis, MO, USA) were added to duplicate wells. Duplicate wells with unstimulated PBMCs were included as background controls (BG). All plates were incubated for 72 h at 37 °C in a humidified 5% CO_2_ atmosphere. After this incubation period, 1 mL of supernatant from duplicate of *Lb*Ag- or PHA-stimulated or unstimulated wells were collected, diluted in 5 mL of Ca^2+^/Mg^2+^-free Hanks’ balanced salt solution (HBSS; Sigma-Aldrich, Cotia, Brazil), and centrifuged at 300× *g* at 20 °C for 10 min to remove cells and large debris [33]. The cleared supernatant was then passed through a 0.8 µm filter (Merck, Darmstadt, German) and spun again at 2000× *g* at 20 °C for 10 min to eliminate dead cells and residual debris. EV were pelleted by centrifugation at 10,000× *g* at 4 °C for 40 min. The resulting pellet was washed with 1 mL annexin V buffer (Anx-B; BD Biosciences, São Paulo, Brazil) at 10,000× *g* for, 4 °C for 40 min, to pellet EV. Then, the pellet was resuspended in Anx-B and subject to the EV labeling protocol for nFCM.

### 2.8. EV Labeling Protocol for Nano-Flow Cytometry

We adapted the nano-flow cytometry (nFCM) staining protocol, previously defined by Costa et al. (2024) [32] to quantify and phenotype EV in supernatant of PBMC cultures (LPA). Prior to the staining, the antibodies/annexin V/calcein mixture was centrifuged at 20,000× *g* for 30 min at 4 °C to remove fluorescent aggregated particles. For labeling EV, an aliquot of the EV-concentrated pellet (100 µL) (see Section 2.7) was incubated with the following monoclonal antibody (mAb) panel: 2.5 µL annexin V-FITC (BD Biosciences, São Paulo, Brazil), 2.5 µL calcein violet AM (BioLegend, San Diego, CA, USA), and 3 µL each of anti-CD4-APC, anti-CD8-PE Cy5.5, and anti-CD14-PE (all Beckman Coulter, Brea, CA, USA) to the 100 µL EV suspension. After 30 min incubation at 4 °C, samples were brought to 2 mL total volume with Anx-B, to avoid swarm effects, and were immediately analyzed by nFCM.

All intra-assay quality controls were applied following the rules defined by MISEV 2023 and MIFlowCyt-EV guidelines [34,35,36]: (a) EV’ size analytic 160–500 nm region was defined gating on Megamix-Plus SSC polystyrene beads (BioCytex, Marseille, France) histogram (Figure 2G); (b) a 0.2 µm-filtered Anx-B-only control; (c) Anx-B plus MoAbs/annexin V/calcein mix, to confirm removal of fluorescent background particles; (d) unstained controls; (e) PE isotype control (Beckman Coulter, São Paulo, Brazil), pre-centrifuged at 20,000× *g* for 30 min; and (f) detergent NP-40 (IGEPAL CA-63, Sigma-Aldrich, St. Louis, MO, USA) EV lysis control, to confirm vesicle detection.

### 2.9. Nano-Flow Cytometry Analysis of Extracellular Vesicles

Flow cytometry was performed using a CytoFLEX flow cytometer (Beckman Coulter, São Paulo, Brazil) equipped with 405 nm, 488 nm, and 638 nm lasers, configured for nano-scale measurements following the protocol previously established by Costa et al. (2024) [32]. In brief, to optimize EV detection, the standard flow cytometry configuration was modified by using 405 nm violet side scatter (VSSC) as the trigger signal instead of the conventional 488 nm forward scatter (FSC). Dual threshold triggering on VSSC and FITC channels was used to minimize background noise. For routine cleaning, the system was flushed with 0.2 µm-filtered Coulter Clenz (Beckman Coulter, São Paulo, Brazil) for 5 min, followed by a 15-min rinse with ultrapure water. Milli-Q water was used as the sheath fluid, and between-sample carryover was prevented with 2-min high-speed rinses using ultrapure water. All samples were acquired at low speed, and acquisition continued for up to 60 min or until sample depletion. Instrument settings were held constant across all samples.

### 2.10. Data Analysis and Statistics

Flow cytometric data were analyzed using CytExpert v2.6 software (Beckman Coulter, São Paulo, Brazil), while statistical analysis was performed using GraphPad Prism version 8.0 (GraphPad Software, San Diego, CA, USA).

To assess the specific impact of antigenic stimulation on EV release, the net change in EV concentration was calculated for each sample. For this purpose, we determined the median EV concentration (EV/µL) in both stimulated and unstimulated wells. The net change was calculated using the following formula:

*Net Change EV = (Median EV/µL in stimulated wells) minus (Median EV/µL in unstimulated wells)*

This approach allowed us to isolate the EV production specifically induced by stimulation, correcting for baseline EV release in unstimulated conditions. Net change values, presented as median with interquartile range (IQR), were subsequently used for group comparisons and statistical analyses.

Statistical analysis was performed with ANOVA followed by nonparametric Mann–Whitney’s post hoc test to compare differences between individual groups. Statistical significance was defined as follows: *p* < 0.05 (*), *p* < 0.01 (**), *p* < 0.001 (***), and *p* < 0.0001 (****).

## 3. Results

### 3.1. Optimization of Extracellular Vesicle Detection in Lymphocyte Proliferative Assay

To enable precise detection and characterization of extracellular vesicles (EV) secreted by peripheral blood mononuclear cells (PBMCs), we established a workflow with multiple methodological refinements aimed at minimizing background noise and enhancing assay specificity. First, blood collection and PBMCs handling procedures were rigorously standardized to prevent ex vivo EV formation. Key steps included discarding the first blood-collected tube, using heparin as anticoagulant, and avoiding mechanical agitation prior to processing.

**Figure 1:**
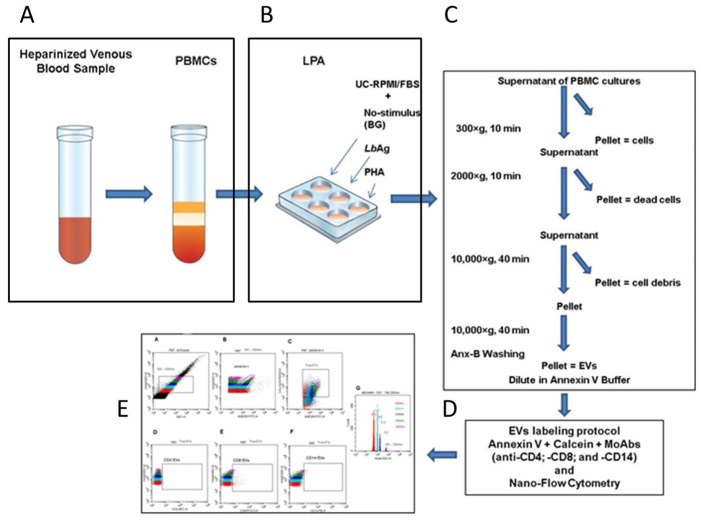
Workflow for extracellular vesicle (EV) isolation and detection in PBMC cultures. Schematic overview of the optimized protocol including: (**A**) blood collection and PBMC isolation with careful handling to prevent artificial EV generation; (**B**) PBMC culture conditions: unstimulated (background, BG), stimulated with *L. (V.) braziliensis* antigen (*Lb*Ag), or with phytohemaglutinin (PHA); (**C**) sequential centrifugation and filtration steps to obtain EV-containing supernatants: to remove cells spin at 300× *g*, to remove dead cells at 2000× *g*, and to exclude debris at 10,000× *g*. The final EV-concentrated pellet, was subjected to another 10,000× *g* spin in annexin V binding buffer (Anx-B); (**D**) EV labeling protocol with annexin V, calcein AM, and anti-CD4, anti-CD8, and anti-CD14 monoclonal antibodies; (**E**) representative nano-flow cytometry (nFCM) gating strategy for EV subset analysis.

Second, to deplete exogenous EV from the culture medium, RPMI 1640 supplemented with fetal bovine serum (FBS) was subjected to ultracentrifugation (100,000× *g* for 18 h) and sterile filtration, generating an EV-depleted medium (UC-RPMI/FBS) that was used throughout cell cryopreservation and lymphocyte proliferation assays (LPA). After stimulation, culture supernatants underwent sequential centrifugation and filtration steps to remove intact cells, dead cells, and debris. EV were pelleted by high-speed centrifugation, resuspended in annexin V binding buffer, and immediately processed for nano-flow cytometry (nFCM) labeling and acquisition using a standardized gating strategy (Figure 1).

### 3.2. Definition of a Nano-Flow Cytometry Gating Strategy for EV Quantification and Phenotyping in PBMC Cultures from CL Patients and Healthy Individuals

For nFCM acquisition, we optimized the labeling protocol by pre-clearing fluorophore aggregates from the monoclonal antibodies/annexin V/calcein mixture by centrifugation at 20,000× *g* for 30 min at 4 °C. Sample acquisition was performed on a CytoFLEX (Beckman Coulter, Fort Collins, Co, USA) flow cytometer configured with violet side scatter (VSSC, 405 nm), dual-threshold triggering, and nanometric calibration using Megamix polystyrene beads. Intra-assay controls included buffer-only, isotype, reagent, and detergent lysis controls to ensure the specificity of EV detection and minimize artifacts.

**Figure 2:**
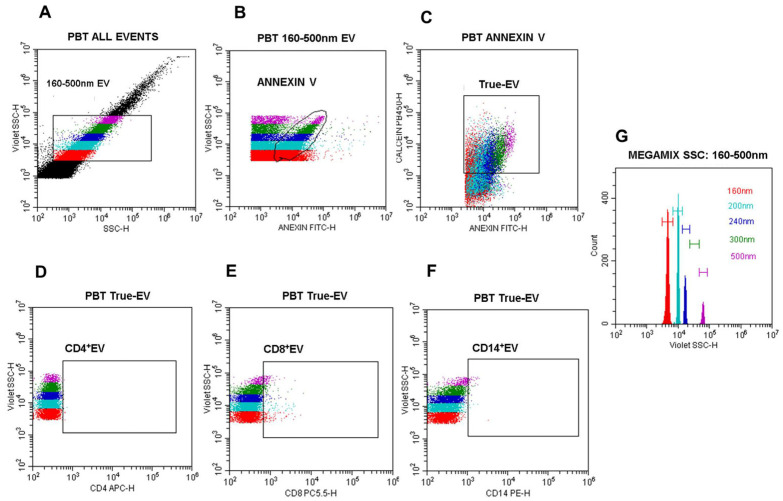
Nano-flow cytometry gating strategy for EV detection and phenotyping. (**A**) Dot plot blue side scatter (SSC)-log-H vs. violet side scatter (VSSC)-log-H: the 160–500 nm EV gate was defined based on Megamix VSSC bead histograms(G); (**B**) dot plot annexin V-FITC-log-H vs. violet SSC-log-H gated on the 160–500 nm region to identify annexin V^+^ EV; (**C**) dot plot annexin V vs. calcein AM gated on annexin V^+^ EV to define calcein^+^/annexin V^+^ (double-positive) vesicles population (True-EV); (**D**) dot plot CD4-APC vs. VSSC-log-H gated on True-EV; (**E**) dot plot CD8-PECy5.5 vs. VSSC-log-H gated on True-EV; (**F**) dot plot CD14-PE vs. VSSC-log-H gated on True-EV; (**G**) histogram of violet SSC-log-H defining the relative diameters of Megamix SSC polystyrene beads (160 nm, 200 nm, 240 nm, 300 nm, 500 nm). All analyses were performed using the CytoExpert software, version 2.6 (Beckman Coulter, São Paulo, Brazil).

The following gating strategy was applied: (A) blue SSC vs. VSSC log scale dot plot to define a 160–500 nm EV population based on Megamix bead calibration (Figure 2G); (B) annexin V-FITC vs. VSSC dot plot gated on the 160–500 nm region to identify phosphatidylserine-positive EV; (C) annexin V vs. calcein AM dot plot gated on annexin V^+^ events to define intact EV (True-EV); (D–F) CD4-APC, CD8-PECy5.5, and CD14-PE vs. VSSC dot plots gated on True-EV for immune subset phenotyping; and (G) VSSC histogram for precise size calibration based on Megamix polystyrene bead standards (Figure 2).

### 3.3. L. (V.) braziliensis Antigen Stimulation Modulates EV Release in PBMC Cultures

We assessed the effect of *L. (V.) braziliensis* antigen (*Lb*Ag) on EV release by calculating the “net change” in EV concentrations, defined as the difference between EV counts in *Lb*Ag-stimulated and unstimulated (background) PBMC cultures for each participant (Supplementary Table S1).

**Figure 3:**
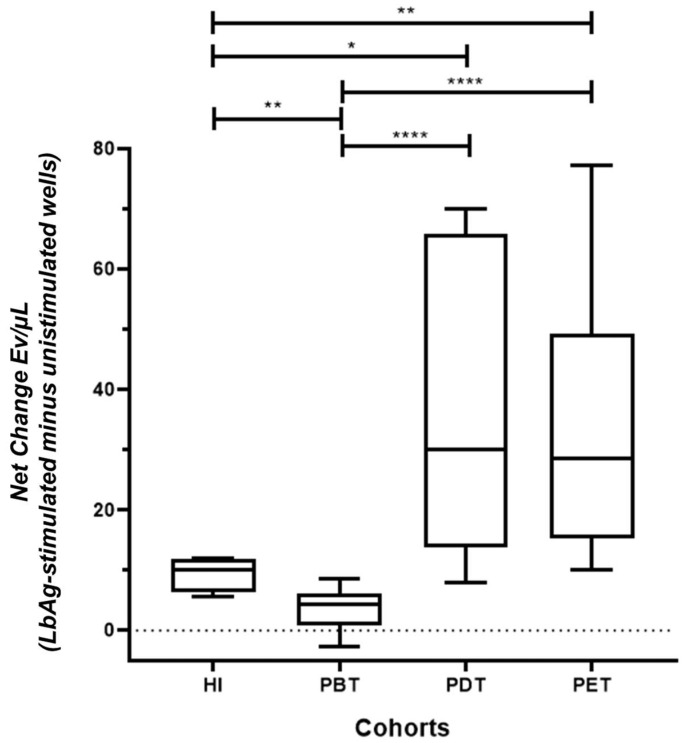
Modulation of total EV concentrations in PBMC cultures from CL patients and healthy individuals in response to *Lb*Ag stimulation. Net change in EV concentrations (EV/µL) in PBMC supernatants following *Lb*Ag stimulation compared to unstimulated controls. Net change was calculated by subtracting EV counts in unstimulated wells from those in stimulated wells for each individual. EV release was significantly suppressed in the pre-treatment (PBT) group compared to healthy individuals (HI) (** *p* = 0.0018). EV levels were significantly elevated in patients during treatment (PDT) and post-treatment (PET) compared to PBT (**** *p* < 0.0001 for both) and similar to HI (* *p* = 0.012; ** *p* = 0.005, respectively). Box plots display median, interquartile range (IQR), and individual data points. Statistical significance was determined using the Mann-Whitney U test.

In the pre-treatment (PBT) group, *Lb*Ag stimulation significantly suppressed total EV release compared to healthy individuals (HI) (** *p* = 0.0018). In contrast, patients during treatment exhibited significantly higher EV release than PBT patients (**** *p* < 0.0001 for both comparisons) and reached levels comparable to HI (* *p* = 0.012 and ** *p* = 0.005, respectively) (Figure 3).

These findings indicate that EV production is actively suppressed during untreated CL, but is markedly restored during and after antimonial therapy, suggesting a close relationship between EV dynamics and treatment response.

### 3.4. LbAg-Induced Changes in CD4^+^, CD8^+^, and CD14^+^ EV Subsets

We further analyzed EV subsets derived from CD4^+^, CD8^+^, and CD14^+^ cells using the same net change calculation (stimulated minus unstimulated). Overall results are shown in Supplementary Table S1.

**Figure 4:**
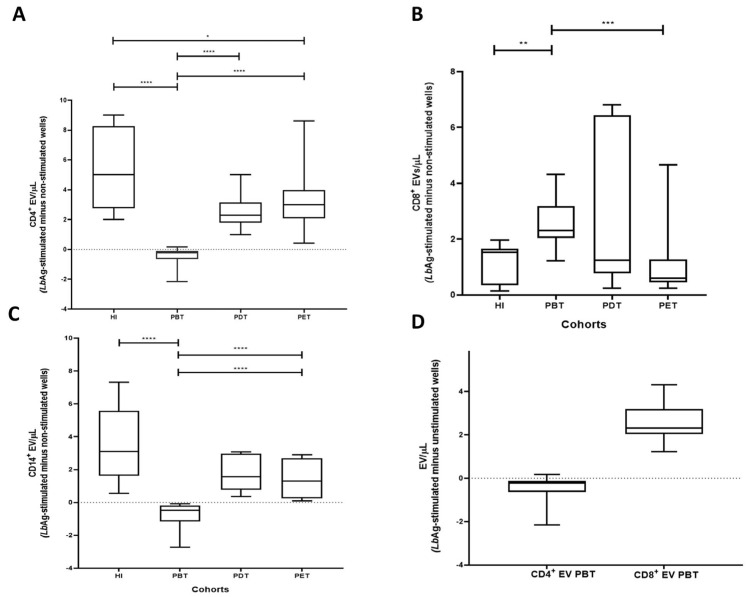
Subset-specific EV profiles in PBMC cultures from CL patients and healthy individuals. (**A**) Net change in CD4^+^ EV concentrations. PBT cultures exhibited significantly reduced CD4^+^ EV compared to HI (**** *p* < 0.0001). CD4^+^ EV were significantly restored in PDT and PET groups compared to PBT (**** *p* < 0.0001) and reached levels comparable to HI (* *p* = 0.03; *p* = 0.23, respectively). (**B**) Net change in CD8^+^ EV concentrations. CD8^+^ EV were elevated in the PBT group compared to HI (** *p* = 0.0012). Levels in PDT and PET were similar to HI (*p* = 0.71 and *p* = 0.63, respectively). PET EV levels were significantly lower than PBT (*** *p* = 0.0004). (**C**) Net change in CD14^+^ EV concentrations. CD14^+^ EV were significantly lower in the PBT group compared to HI (**** *p* < 0.0001). EV levels in PDT and PET groups were significantly higher than in PBT (**** *p* < 0.0001) and similar to HI (*p* = 0.115 and *p* = 0.061, respectively). (**D**) Comparative analysis within the PBT group revealed significantly higher CD8^+^ EV than CD4^+^ EV (**** *p* < 0.0001). Box plots display median, IQR, and individual data points. Statistical comparisons were performed using the Mann–Whitney U test.

In the PBT group, CD4^+^ EV levels (EV/µL) were significantly reduced compared to HI (**** *p* < 0.0001). In contrast, CD4^+^ EV concentration ranks significantly increased in PDT and PET compared to PBT (**** *p* < 0.0001) and approached levels observed in HI (* *p* = 0.03 and *p* = 0.23, respectively) (Figure 4A).

The PBT group exhibited significantly higher CD8^+^ EV levels compared to HI (** *p* = 0.0012). Otherwise, CD8^+^ EV levels in PDT and PET were not significantly different from HI (*p* = 0.71 and *p* = 0.63, respectively). Although PDT levels remained similar to PBT (*p* = 0.43), CD8^+^ EV were significantly lower in PET compared to PBT (*** *p* = 0.0004) (Figure 4B).

CD14^+^ EV levels were significantly decreased in the PBT group compared to HI (**** *p* < 0.0001). Levels in PDT and PET were not significantly different from HI (*p* = 0.115 and *p* = 0.061, respectively), but were significantly higher than in PBT (**** *p* < 0.0001 for both) (Figure 4C).

Within the PBT group, comparative analysis of EV subsets revealed that CD8^+^ EV concentrations were significantly higher compared to CD4^+^ EV levels (**** *p* < 0.0001) (Figure 4D).

Collectively, these findings indicate that acute CL is associated with a skewed EV profile, characterized by reduced CD4^+^ and CD14^+^ EV and elevated CD8^+^ EV levels, while treatment appears to modulate the release of these EV subsets to physiologic pattern.

## 4. Discussion

This study provides novel insights into the immunopathogenesis of human cutaneous leishmaniasis (CL), demonstrating that *Leishmania (Viannia) braziliensis* distinctly modulates the release of extracellular vesicles (EV), including those derived from CD4^+^, CD8^+^, and CD14^+^ immune cells. By integrating nano-flow cytometry (nFCM) with a modified lymphocyte proliferation assay (LPA), we established a robust platform to quantify and phenotype EV released in PBMC cultures from patients at different clinical stages: acute disease (PBT), during treatment (PDT), after clinical cure (PET), and from healthy individuals (HI). Distinct patterns of EV release were observed under both unstimulated and *L. (V.) braziliensis* antigen (*Lb*Ag)-stimulated conditions, correlating with disease activity and therapeutic response.

Our findings support the hypothesis that EV mirror the host immune activation state and may serve as sensitive biomarkers for treatment monitoring in CL. This is consistent with findings in other infectious diseases, where EV play central roles in immune modulation, antigen presentation, and disease surveillance [37,38,39,40].

Several methodological advances underpinned the reliability and resolution of our EV data. While traditional EV characterization techniques, such as nanoparticle tracking analysis (NTA), dynamic light scattering (DLS), and transmission electron microscopy (TEM) have limited capacity to phenotype vesicles at the single-particle level [27,41], nFCM enables concurrent detection and characterization of individual vesicles in the nanometer range.

The use of violet side scatter (VSSC, 405 nm) improved resolution and sensitivity for small particles [42,43], overcoming some of the limitations of conventional flow cytometry in EV research. Additionally, to ensure data robustness, we applied stringent pre-analytical procedures. These included ultracentrifugation and filtration of the culture medium to eliminate exogenous vesicles, as well as sequential centrifugation steps to minimize cellular debris. Moreover, we implemented careful handling protocols to reduce artificial vesicle formation—a known source of experimental noise. A key methodological advancement was the dual fluorescent labeling strategy using annexin V and calcein AM. While annexin V binds phosphatidylserine (PS) on vesicle membranes, it cannot distinguish intact vesicles from apoptotic debris. Calcein AM, on the other hand, fluoresces only within intact vesicles. By selecting for double-positive events, we increased the specificity of EV identification, ensuring that only intact vesicles (“True-EV”) were included in the analysis [33,44]. This combined strategy strengthened the reliability of EV quantification and minimized the likelihood of misinterpreting membrane debris as functional vesicles.

Functionally, we observed a reduction in total and subset-specific EV (CD4^+^, CD8^+^, and CD14^+^) in *Lb*Ag-stimulated PBMC cultures from untreated CL patients. This pattern diverges from what is typically seen in viral infections, where pathogen-derived stimuli enhance EV production [23]. In CL, this suppression may result from active inhibition of host vesicle biogenesis or secretion pathways by *L. (V.) braziliensis*. Previous studies have shown that *Leishmania* parasites can manipulate host endosomal trafficking, vesicle release, and antigen presentation to evade immune responses [25]. Therefore, the downregulation of EV release observed here—especially from CD4^+^ and CD14^+^ cells—may represent a parasite-driven immune evasion mechanism during the acute phase of CL. Notably, our study did not assess the frequency of immune cell subsets in parallel with EV quantification, and therefore we cannot rule out that changes in EV levels may be partially attributed to variations in the frequencies of CD4^+^, CD8^+^, or CD14^+^ cells in culture.

Remarkably, CD8^+^ EV remained relatively elevated even in untreated patients (PBT), potentially reflecting persistent cytotoxic T cell activity. This observation aligns with studies implicating CD8^+^ T cells in both protective and pathogenic roles in CL [10,11,12,13,14,15,16,17].

Upon antimonial treatment, we observed a progressive restoration of EV levels across all subsets, often exceeding those found in healthy controls. This increase likely reflects immune reactivation following parasite burden reduction, as previously reported with meglumine antimoniate therapy [5].

A marked increase in CD4^+^ and CD8^+^ EV was observed, suggesting partial recovery of T cell function. The restoration of CD4^+^ EV may reflect renewed Th1 cytokine production and antigen-presenting cell (APC) activity, while sustained CD8^+^ EV likely indicate ongoing effector function. Additionally, CD14^+^ EV exhibited partial increase, pointing to the reactivation of monocyte/macrophage involvement in immune signaling and antigen presentation [45,46].

The persistence of elevated EV from all three subsets in clinically cured patients (PET) raises the possibility that EV contribute to long-term immune surveillance or residual antigen clearance. CD4^+^ EV may help maintain Th1 polarization, while CD8^+^ and CD14^+^ EV may support cytotoxic readiness and innate responses within lymphoid niches.

Taken together, our findings reveal a biphasic modulation of EV during CL: suppression during active infection, followed by restoration with treatment. Monitoring immune cell–derived EV, especially CD4^+^ and CD14^+^ subsets, may provide valuable insights into disease progression and therapeutic efficacy, complementing traditional parasitological and cytokine-based assessments.

Some limitations should be considered when interpreting the findings: (i) Although 37 CL patients were enrolled, the number of individuals in each clinical subgroup (PBT, PDT, PET) was relatively small (10–17), limiting statistical power and the generalizability of subgroup-specific conclusions. (ii) The absence of longitudinal follow-up in the same individuals precludes the assessment of individual EV dynamics over the course of infection and treatment. This approach would provide more robust insights into the dynamics of EV release and its potential as a biomarker of therapeutic response. (iii) Another important limitation is the lack of parallel quantification of CD4^+^, CD8^+^, and CD14^+^ cell frequencies in the PBMC cultures. Without this information, it is not possible to discern whether differences in EV levels reflect true modulation of vesicle release or simply shifts in the number of each immune cell subset. (iv) The exclusive use of PBMCs does not capture the full complexity of the cutaneous microenvironment where *Leishmania* infection occurs. Tissue-resident cells such as keratinocytes, fibroblasts, endothelial cells, and dermal macrophages likely contribute significantly to EV production and immune modulation, and future studies incorporating lesion biopsies or skin-derived models would offer a more physiologically relevant perspective on local immune responses. (v) While the use of VSSC-enhanced nFCM mitigates size estimation biases, the reliance on calibration beads with higher refractive indices than biological vesicles remains an inherent limitation in flow cytometry–based EV studies [34,35,36,41]. (vi) The absence of clinical metadata (e.g., lesion size, disease duration) and cytokine profiles limits the ability to contextualize EV patterns in terms of disease severity and immune status. Integrating these parameters in future investigations would strengthen the immunopathogenic interpretation and the potential translational relevance of EV as biomarkers.

In conclusion, this study highlights the potential of immune cell–derived EV as biomarkers of immune dynamics in human cutaneous leishmaniasis. We identified a biphasic pattern of EV release—initial suppression during active infection followed by restoration during and after therapy—most notably affecting the CD4^+^ and CD14^+^ subsets. While CD8^+^ EV remained relatively elevated throughout, possibly reflecting cytotoxic activity, the observed trends suggest that EV mirror the host immune status and treatment response. By employing refined nano-flow cytometry and a dual-labeling strategy, we developed a reproducible platform for EV phenotyping directly from PBMC cultures. These findings provide a foundation for future longitudinal and tissue-based studies to further elucidate the role of EV in CL immunopathogenesis and cure.

## Figures and Tables

**Table 1:** Characteristics of the 37 cutaneous leishmaniasis (CL) patients and 6 healthy individuals enrolled in this study.

	Cohorts			
Variable	PBT	PDT	PET	HI
(*n*)	17	10	10	6
Sex				
Female	7	5	5	3
Male	10	5	5	3
Age (mean±SD)	34.06 ± 14.25	42.83 ± 15.60	31.60 ± 14.40	29.6 ± 10.10

**PBT:** patients before treatment; **PDT:** patients during treatment; **PET**: patients at the end of treatment (clinically cured); **HI**: healthy individuals. SD: standard deviation.

## Data Availability

All research data is available by the authors on request.

## Supplementary Materials

The following supporting information can be downloaded at: https://www.mdpi.com/article/10.3390/pathogens14080771/s1, Supplementary Table S1: Concentrations of Total EV and Immune cell–derived EV in PBMC cultures from CL patients and healthy individuals following *L. (V.) braziliensis* antigen stimulation.

## Abbreviations

The following abbreviations are used in this manuscript:

nFCM	Nano-flow cytometry
LPA	Lymphocyte lymphoproliferative assay
EV	Extracellular vesicles
PBMCs	Peripheral blood mononuclear cells
*Lb*Ag	*Leishmania (V.) braziliensis* antigens
Anx-B	Annexin V buffer
