# Supplementary material for: Extracellular Vesicle Release from Immune Cells in Cutaneous Leishmaniasis: Modulation by Leishmania (V.) braziliensis and Reversal by Antimonial Therapy
Source: Pathogens. 2025 Aug 4;14(8):771. doi: 10.3390/pathogens14080771 (PMC12389337; doi:10.3390/pathogens14080771)
Supplement: Supplementary file 1 [file pathogens-14-00771-s001.zip › pathogens-3765761-supplementary.pdf]

Table S1. Concentrations of Total EVs and Immune cell–derived EVs in PBMC cultures from CL patients and healthy individuals following *L. (V.) braziliensis* antigen stimulation.

	**PBT (*n* = 17)**	**PDT (*n* = 10)**	**PET (*n* = 10)**	**HI (*n* = 6)**
Total EVs	-4.36 [-8.60 – -2.70]	30.01 [8.00 – 86.41]	28.66 [10.00 – 87.04]	10.16 [5.60 – 12.00]
$CD4^+$ EVs	-0.23 [-2.16 – 0.17]	2.28 [0.98 – 5.00]	3.00 [0.42 – 8.60]	5.00 [2.00 – 9.00]
$CD8^+$ EVs	2.32 [1.23 – 4.32]	1.25 [0.24 – 6.81]	0.61 [0.24 – 4.67]	1.53 [0.15 – 1.96]
$CD14^+$ EVs	-0.48 [-2.70 – -0.07]	1.58 [0.37 – 3.08]	1.30 [0.10 – 2.91]	3.11 [0.55 – 7.32]

Values are presented as *Net Change* EVs/μL: median and [interquartile range (IQR)] of Stimulated minus Unstimulated PBMCs culture wells; **PBT**: Patients before treatment (acute phase); **PDT**: Patients during treatment; **PET**: Patients at the end of treatment (clinically cured); **HI:** Healthy individuals.
